# The Avidity of Autoreactive Alpha-Synuclein Antibodies in Leucine-Rich Repeat Kinase 2 Mutation Carriers Is Not Altered Compared to Healthy Controls or Patients with Parkinson’s Disease

**DOI:** 10.3390/biom13091303

**Published:** 2023-08-25

**Authors:** Alexandra Albus, Yannick Kronimus, Monika Burg-Roderfeld, Hendrik van der Wurp, Dieter Willbold, Tamar Ziehm, Richard Dodel, Jean Alexander Ross

**Affiliations:** 1 Therapy Research in Neurogeriatrics, Center for Translational Neuro- and Behavioral Sciences, University of Duisburg-Essen, University Hospital Essen, 45147 Essen, Germany; alexandra.albus@uk-koeln.de (A.A.); yannickk89@web.de (Y.K.); vanderwurp@statistik.tu-dortmund.de (H.v.d.W.); alexander.ross@uni-due.de (J.A.R.); 2 Department for Neurology, Philipps-University Marburg, 35037 Marburg, Germany; 3 Department of Chemistry and Biology, Fresenius University of Applied Sciences, 65510 Idstein, Germany; monika.burg-roderfeld@hs-fresenius.de; 4 Faculty of Statistics, TU Dortmund University, 44227 Dortmund, Germany; 5 Institute of Physical Biology, Heinrich Heine University Düsseldorf, 40225 Düsseldorf, Germany; d.willbold@fz-juelich.de (D.W.);; 6 Institute of Biological Information Processing (IBI-7: Structural Biochemistry), Forschungszentrum Jülich, 52428 Jülich, Germany

**Keywords:** alpha-synuclein, naturally occurring autoantibodies, Parkinson’s disease, LRRK2, PARK8

## Abstract

The accumulation and aggregation of alpha-synuclein (α-Syn) are pathological processes associated with Parkinson’s disease, indicating that the regulation of protein is a crucial etiopathological mechanism. Interestingly, human serum and cerebrospinal fluid contain autoantibodies that recognize α-Syn. This potentially demonstrates an already existing, naturally decomposing, and protective system. Thus, quantitative or qualitative alterations, such as the modified antigen binding of so-called naturally occurring autoantibodies against α-Syn (nAbs-α-Syn), may induce disease onset and/or progression. We investigated the serum titers and binding characteristics of nAbs-α-Syn in patients suffering from sporadic Parkinson’s disease (n = 38), LRRK2 mutation carriers (n = 25), and healthy controls (n = 22). Methods: Titers of nAbs-α-Syn were assessed with ELISA and binding affinities and kinetics with SPR. Within the patient cohort, we discriminated between idiopathic and genetic (LRRK2-mutated) variants. Results: ELISA experiments revealed no significant differences in nAbs-α-Syn serum titers among the three cohorts. Moreover, the α-Syn avidity of nAbs-α-Syn was also unchanged. Conclusions: Our findings indicate that nAbs-α-Syn concentrations or affinities in healthy and diseased persons do not differ, independent of mutations in LRRK2.

## 1. Introduction

Parkinson’s disease (PD) is the second most common neurodegenerative disorder and the most common movement disorder. One of its pathological hallmarks relates to the intracellular deposits of alpha-synuclein (α-Syn) protein, forming Lewy bodies and Lewy neurites in the neurons of the *substantia nigra pars compacta*. The protein α-Syn, apart from its pivotal role in synaptic transmission, exhibits various neurotoxic traits when aggregated, contributing to neurodegeneration and giving rise to hallmark clinical symptoms (such as bradykinesia, rigor, and tremor) [1].

While the majority of PD cases occur sporadically, up to 5% of all individuals with Parkinson’s disease (PwP) exhibit a genetic background [2]. Among these cases, mutations within the PARK8 gene, encoding the serine–threonine kinase leucine-rich repeat kinase 2 (LRRK2), account for a substantial portion and are inherited through an autosomal dominant pattern [3]. Though distinguishing clinically between sporadic PD and LRRK2-associated PD remains challenging, considerable evidence indicates a comparatively slower progression in the latter [4]. Furthermore, the spectrum of neuropathological observations varies considerably, including instances devoid of Lewy bodies. This variability may reflect heterogeneous contributions of LRRK2, contingent on specific mutations, to the pathological cascade. The aberrant phosphorylation of α-Syn, co-regulation, the initiation of proinflammatory cytokine release, and mitochondrial fragmentation represent just a few of the proposed links between LRRK2 and PD-related pathology [5,6].

To date, no reliable early biomarkers or successful therapeutic strategies for a cure have emerged for PD. In order to address this problem, we focus on investigating naturally occurring autoantibodies (nAbs) against α-Syn (nAbs-α-Syn). These nAbs, deemed part of the body’s first line of defense, allow physiological homeostasis to be maintained [7]. Originating from B cells in a manner similar to murine B-1 cells, their analogy in humans continues to be debated [8,9]. We hypothesize that nAbs-α-Syn possesses a regulatory function to α-Syn proteostasis, potentially exhibiting qualitative or quantitative alterations as etiopathological events in PD.

In the present study, we investigated nAbs-α-Syn avidity and serum titers in control subjects, patients suffering from sporadic PD, and carriers of LRRK2 mutations. Through this examination, we aim to uncover any divergences between the groups, thus shedding light on potential underlying pathological mechanisms.

## 2. Materials and Methods

### 2.1. Participants

Serum samples of 22 control subjects, 38 sporadic (non-LRRK2 mutation carriers) PwP, and 25 LRRK2 mutation carriers were provided by the MJFF-sponsored LRRK2 Cohort Consortium (LCC). For up-to-date information on the study, visit www.michaeljfox.org/lcc (accessed on 23 July 2023). All participants were age- and gender-stratified, as outlined in Table 1.

### 2.2. Proteins and Antibodies

Alpha-synuclein (α-Syn, Analytik Jena GmbH, Jena, Germany) was provided in a 5 mg/mL stock solution and used in its monomeric form. For ELISA experiments, nAbs-α-Syn samples were purified from intravenous immunoglobulins of class G (IVIg; Octapharma GmbH, Langenfeld, Germany), as previously described and used as an internal standard [10]. A biotinylated goat anti-human IgG (Dianova, Hamburg, Germany) was used as a secondary antibody. For SPR analyses, the monoclonal anti-α-Syn antibody 211 (Santa Cruz, Dallas, TX, USA) was used as an internal control.

### 2.3. IgG Purification

First, serum IgG was purified using 200 µL of serum and the Melon™ Gel Purification Kit (Thermo Fisher Scientific, Waltham, MA, USA), in line with the manufacturer’s instructions. Due to the incompatibility of the Melon buffer for SPR experiments [11], a buffer exchange into PBS using Vivaspin columns (Vivaspin6, 50,000 MWCO, Sartorius, Göttingen, Germany) and a subsequent lyophilization step (Alpha 1-4, Martin Christ Gefriertrocknungsanlagen GmbH, Osterode am Harz, Germany) were performed. Afterwards, all samples were resuspended in H_2_O to the initial volume (200 µL). The IgG concentration was measured using a NanoDrop™ ND-1000 spectrometer (Thermo Scientific, Waltham, MA, USA).

### 2.4. Determination of nAbs-α-Syn within Total IgG Pool

In order to compare the nAbs-α-Syn binding characteristics investigated by SPR, it is important that for each participant, identical and constant nAbs concentrations were applied. Thus, investigating the active fraction of nAbs-α-Syn within the IgG pool of each subject was the first step determined by ELISA experiments. Thus, 96-well plates were coated with 3 µg/mL of α-Syn in PBS and incubated for 24 h at 4 °C. Afterwards, plates were blocked with 1× Roti-block (Carl Roth GmbH, Karlsruhe, Germany) + 0.1% Tween 20 (AppliChem GmbH, Darmstadt, Germany) for 1 h at 37 °C. IgG samples were added in triplicate and with four different dilutions to both α-Syn- and PBS-coated wells. Furthermore, each ELISA plate contained the nAbs-α-Syn standard, covering a broad concentration range. The ELISA was developed by adding a biotinylated goat anti-human IgG antibody (1:20,000), streptavidin-conjugated peroxidase (1:200; R&D Systems, Minneapolis, MN, USA), its substrate TMB (tetramethylbenzidine; Merck KGaA, Darmstadt, Germany), and 5% sulfuric acid (Carl Roth GmbH, Karlsruhe, Germany) as the stop solution. The OD of the resulting color reaction was measured at 450 nm using a plate reader (Infinite M200, Tecan, Männedorf, Switzerland). With the aid of the autoantibody standard, the nAbs-α-Syn concentration of each serum sample was calculated.

### 2.5. SPR Measurements

SPR experiments were performed on the BIAcore T200 (GE Healthcare, Chicago, IL, ISA) protein interaction array system at 25 °C. The α-Syn protein was dissolved in a 10 mM acetate buffer (pH 4.0; GE Healthcare, Chicago, IL, USA) and was covalently immobilized onto a flow cell of a CM5 sensor chip (GE Healthcare, Chicago, IL, ISA) via amine coupling, according to the manufacturer’s protocol, with 1400 response units (RUs). To verify successful and constant protein immobilization over time, the monoclonal antibody 211 (7.5 µg/mL) was used as an internal control before each measurement day. One channel was used as the reference channel and remained uncoated. The remaining active sites of the sensor chip were blocked after they were treated with 1 M ethanolamine-HCl (pH 8.5; GE Healthcare, Chicago, IL, ISA). PBS with 0.005% Tween 20 (PBST) was used as the running buffer. Interactions were tested at a flow rate of 30 μL/min and a 4 min contact time, followed by 3 min of dissociation. After the interaction experiment was complete, the chip surface was regenerated by injecting 0.85% phosphoric acid at a flow rate of 10 μL/min and a contact time of 30 s.

In order to investigate the binding characteristics of nAbs-α-Syn, IgG samples were measured in a 1:2 downward concentration pattern; the initial IgG concentration was determined using the ELISA results and was calculated to be the IgG concentration containing 3 µg/mL of nAbs-α-Syn.

### 2.6. Analysis

Analyses were performed with the statistics program R [12]. The *ka* (association constant), *kd* (dissociation constant) and *KD* (antibody affinity) values were tested for the null hypothesis that observations from the different groups (LRRK2, PD, and control groups) could originate from the same population. As no distribution assumptions could have been made, a nonparametric approach was chosen. The Kruskal–Wallis test (one-way ANOVA based on ranks) indicated that none of the variables *ka* (*p* = 0.1956), *kd* (*p* = 0.4863), and *KD* (*p* = 0.9976) had significant differences and that the grouped samples could originate from the same population. With the given *p*-values as above, no adjustments were made to the multiple tests, and no post hoc tests were deemed necessary. This was substantiated by the visualization stage using boxplots.

## 3. Results

The aim of this study was to determine the nAbs-α-Syn serum concentrations and binding characteristics of control subjects (CTRs) and PwP with and without a LRRK2 mutation (PwP-LRRK2). We therefore executed ELISA and SPR experiments with purified IgG fractions.

### 3.1. Cohorts Exhibit Equal IgG and nAbs-α-Syn Serum Concentrations

**Figure 1:**
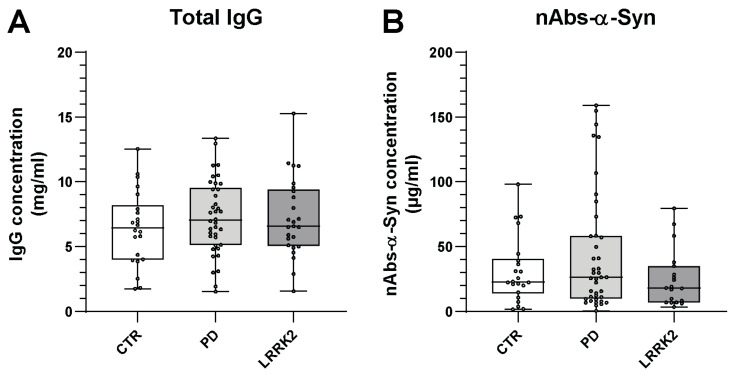
Patient characteristics and nAbs-α-Syn titers. (**A**) Distribution of the determined IgG and (**B**) nAbs-α-Syn concentrations in the three cohorts. Boxplots: 25 and 75 percent quartiles as well as median and mean values (black square); whiskers: min and max values; asterisks: outliers.

In total, 22 CTRs, 38 PwP, and 25 PwP-LRRK2 were included in our study. The participant characteristics are outlined in Table 1. First, the IgG concentration of each subject was determined after purifying the IgG fraction from the corresponding serum sample. Between the three groups, no significant differences were detected, although the control group showed a slightly reduced level of IgG (CTRs: 6.24 ± 2.79 mg/mL; PD patients: 7.27 ± 2.86 mg/mL; and LRRK2 carriers: 7.11 ± 3.06 mg/mL) (Figure 1A, Table 2).

For the following ELISA experiments, IgG samples were diluted in an appropriate manner and added to both α-Syn-coated and uncoated wells. After subtracting the blank signal (uncoated wells) from the specific signal (α-Syn-coated wells), the nAbs-α-Syn concentrations of the study participants were calculated using the nAbs standard under single dilutions. As already seen for IgG, the nAbs-α-Syn concentrations did not significantly differ between the three groups (CTRs: 35.74 ± 25.05 µg/mL; PwP: 47.71 ± 43.90 µg/mL; and PwP-LRRK2: 30.88 ± 31.76 µg/mL) (Figure 1B, Table 2). Although PwP seemed to have higher nAbs concentrations, it is obvious that the levels remarkably vary between the participants within the groups, as indicated by the high standard deviations.

### 3.2. nAbs-α-Syn from the Different Groups Exhibit Equal Avidity

After determining the nAbs-α-Syn concentration in the IgG pool for each study participant, SPR analyses were conducted to investigate individual binding characteristics of the autoantibodies. We therefore analyzed nAbs-α-Syn in a downward concentration pattern, starting at 3 µg/mL, by applying the total IgG containing the corresponding autoantibody concentration. Before each measurement day, the monoclonal antibody 211 was used to verify that there were stable peptide immobilization conditions over time.

**Figure 2:**
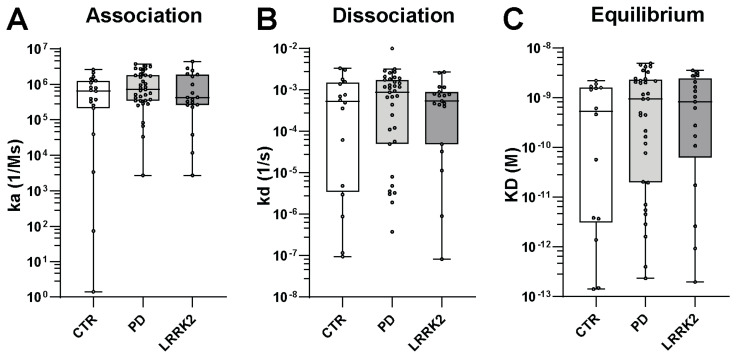
nAbs-α-Syn binding characteristics. (**A**) Calculated association and (**B**) dissociation rates of interactions, separated for control subjects (CTRs) and Parkinson’s patients with (LRRK2) and without (PD) a LRRK2 mutation. (**C**) KD value variations of each group. For statistical analysis, the Kruskal-Wallis test (one-way ANOVA based on ranks) was utilized. Boxplots: 25 and 75 percent quartiles as well as median and mean values (black squares); whiskers: min and max values; asterisks: outliers.

After referencing the interaction curves, the remaining signals showed low RUs in all three cohorts (Figure S1). Due to low signals or buffer discrepancies (between the running buffer and samples), some interactions could not be evaluated. Therefore, 19 CTRs, 38 PwP, and 20 PwP-LRRK2 were used for further analysis. With 1.55 nM for CTR, 1.19 nM for PwP, and 1.2 nM for PwP-LRRK2 (median values), the calculated KD values in all investigated patient groups were similarly distributed (Figure 2; *p* = 0.9976).

## 4. Discussion

In this study, we investigated the potential differences in the titers and binding characteristics of nAbs-α-Syn between healthy people and PwP with and without a LRRK2 mutation. Given the real-time insights into antibody interactions that it provides, we chose surface plasmon resonance (SPR) technology over ELISA measurements to investigate antibody binding, as it allows more detailed conclusions. However, SPR analysis was not able to provide added value, as no significant differences were observed during the interaction phase between the nAbs-α-Syn concentrations of CTRs, PwP, and PwP-LRRK2. Across all cohorts, the affinity of nAbs-α-Syn remained uniform, exhibiting a comparable antibody affinity (KD) in both a general sense and within the details of their associations and dissociations. In contrast, previous investigations that assessed nAbs levels between different cohorts performed ELISA or binding experiments without the preliminary step of IgG purification [13]. Therefore, ELISA results from prior studies could show differences in binding levels due to serum noise signals, which could wrongly reflect lower nAbs-α-Syn titers. To avoid major noise signals, we added the IgG purification step to enable the same conditions for experimental measurements. Nevertheless, the inherent challenge of analyzing nAbs-α-Syn characteristics remained, given their relatively low share within total IgG and IVIg pools.

As nAbs are secreted by innate immune B cells, there is mounting evidence suggesting their impairment during the progression of PD [10,14,15]. Hence, potential quantitative or qualitative nAbs-α-Syn alterations in PD have become relevant, potentially reflecting the underlying pathology. Within the scope of this study, one aim was to elucidate the potential differences of nAbs-α-Syn characteristics among LRRK2 mutation carriers, PwP without this mutation, and CTR participants. This led to the presumption that a LRRK2 mutation can exert an influence on those B cells. However, prior mouse studies demonstrated a lack of LRRK2 expression in B1 cells [16]. This indicates that the development of B1-cell-derived nAbs abnormalities could occur independently from LRRK2 mutations. Furthermore, no differences were observed for nAbs-α-Syn between LRRK2 mutation carriers and PD patients, thereby supporting the initial hypothesis. Nevertheless, similarity in KD values between the control group (CTR) and PD samples offers no definitive substantiation for nAbs-α-Syn avidity as a prospective biomarker for the future.

## 5. Limitation of the Study

For purifying IgG, the entire serum sample from each patient was used. However, as samples were limited to approximately 200 µL, subsequent SPR experiments were performed with the highest nAbs concentration detectable in each sample to allow for comparisons. Therefore, a maximum concentration of 3 µg/mL of nAbs-α-Syn could be used, which is a remarkably low concentration (e.g., in comparison to 7.5 µg/mL of monoclonal antibody or 150 µg/mL of pure nAbs). Thus, this is very likely responsible for the low RUs in the SPR experiments. In addition, the percentage of nAbs-α-Syn in the total amount of IgG was quite low in general, so the signal-to-noise ratio can also be seen as a limiting factor in this study. This study was conducted to analyze qualitative differences in the binding capacity of nAbs-α-Syn. Therefore, the samples were prepared to equalize the nAbs-α-Syn concentration. This changed the concentration of other IgG in the samples, which might influence the binding characteristics of nAbs-α-Syn in the SPR experiments. Importantly, we identified a unique α-Syn bond using nAbs-α-Syn, and no further IgG in these samples seemed to detect the epitope [17].

## 6. Conclusions

Until now, there has been no diagnostic biomarker assay available to diagnose Parkinson’s disease. Given the inconclusive and conflicting outcomes from prior investigations into nAbs-α-Syn serum levels among healthy individuals and patients with the disease, we conducted a comprehensive examination using surface plasmon resonance (SPR) to analyze purified IgG. Our objective was to discriminate between Parkinson’s disease patients, i.e., those with and without a LRRK2 mutation, and healthy subjects. In this study, we observed no significant differences, both in the ELISA experiments and in the main SPR assay. Notably, outcomes across all three cohorts exhibited consistency, thus precluding the development of an nAbs-α-Syn-based biomarker within the constraints of the experimental design presented in this study.

## Figures and Tables

**Table 1:** Characteristics of control subjects (CTRs) and Parkinson’s patients with (LRRK2) and without (PD) a LRRK2 mutation.

					*p*-Value	
	CTR	PD	LRRK2	CTR vs. PD	CTR vs. LRRK2	PD vs. LRRK2
**Sex**						
(m/f)	22 (11/11)	38 (14/24)	25 (14/21)	0.319	0.681	0.134
**Age**						
Mean ± SD	57.1 ± 17.1	64.4 ± 10.2	65.1 ± 7.7	0.078	0.052	0.771
Min–Max	31–85	41–82	43–82			
**Age at Disease Onset**						
Mean ± SD		58.0 ± 9.5	55.5 ± 8.2			0.283
Min–Max		33–74	39–76			

**Table 2:** Characteristics of patient samples and nAbs-α-Syn binding. Determined IgG and nAbs-α-Syn concentrations and median association (ka), dissociation (kd) and equilibrium (KD) values of each cohort.

					*p*-Value	
	CTR	PD	LRRK2	CTR vs. PD	CTR vs. LRRK2	PD vs. LRRK2
**IgG Concentration** (mg/mL)	6.24 ± 2.79	7.27 ± 2.86	7.11 ± 3.06	0.176	0.474	0.633
**nAbs Concentration** (mg/mL)	35.74 ± 25.05	47.71 ± 43.90	30.88 ± 31.76	0.638	0.203	0.231
					***p*-Value**	
				**One-way ANOVA Based on Ranks**		
**ka** (1/Ms)	6.43 × 10^5^	7.00 × 10^−4^	1.55 × 10^−9^		0.1956	
**kd** (1/s)	8.12 × 10^5^	1.15 × 10^−3^	1.19 × 10^−9^		0.4863	
**KD** (M)	4.25 × 10^5^	6.37 × 10^−4^	1.19 × 10^−9^		0.9976	

## Data Availability

The data supporting the findings of this study are available on request from the corresponding author.

## Supplementary Materials

The following supporting information can be downloaded at: https://www.mdpi.com/article/10.3390/biom13091303/s1. Figure S1: nAbs-α-Syn binding characteristics.
