# Supplementary material for: The Avidity of Autoreactive Alpha-Synuclein Antibodies in Leucine-Rich Repeat Kinase 2 Mutation Carriers Is Not Altered Compared to Healthy Controls or Patients with Parkinson’s Disease
Source: Biomolecules. 2023 Aug 25;13(9):1303. doi: 10.3390/biom13091303 (PMC10526238; doi:10.3390/biom13091303)
Supplement: Supplementary file 1 [file biomolecules-13-01303-s001.zip › biomolecules-2548789-supplementary.pdf]

biomolecules

MDPI

Brief Report

Avidity of Autoreactive Alpha-Synuclein Antibodies in LRRK2 Mutation Carriers is Not Altered Compared to Healthy Controls or PD Patients

Alexandra Albus[1,2†], Yannick Kronimus[1,2†], Monika Burg-Roderfeld[3], Hendrik van der Wurp[1,4], Dieter Willbold[5,6], Tamar Ziehm[5], Richard Dodel[1,2,*], J. Alexander Ross[1]

1 Therapy Research in Neurogeriatrics, Center for Translational Neuro- and Behavioral Sciences, University of Duisburg-Essen, Essen, Germany
2 Department for Neurology, Philipps-University, Marburg, Germany
3 Department of Chemistry and Biology, Fresenius University of Applied Sciences, Idstein, Germany
4 Faculty of Statistics, TU Dortmund University, Dortmund, Germany
5 Institute of Physical Biology, Heinrich Heine University Düsseldorf, Düsseldorf, Germany
6 Institute of Biological Information Processing (IBI-7: Structural Biochemistry), Forschungszentrum Jülich, Jülich, Germany
* Correspondence: richard.dodel@uk-essen.de; Tel.: +49 (201) 89760

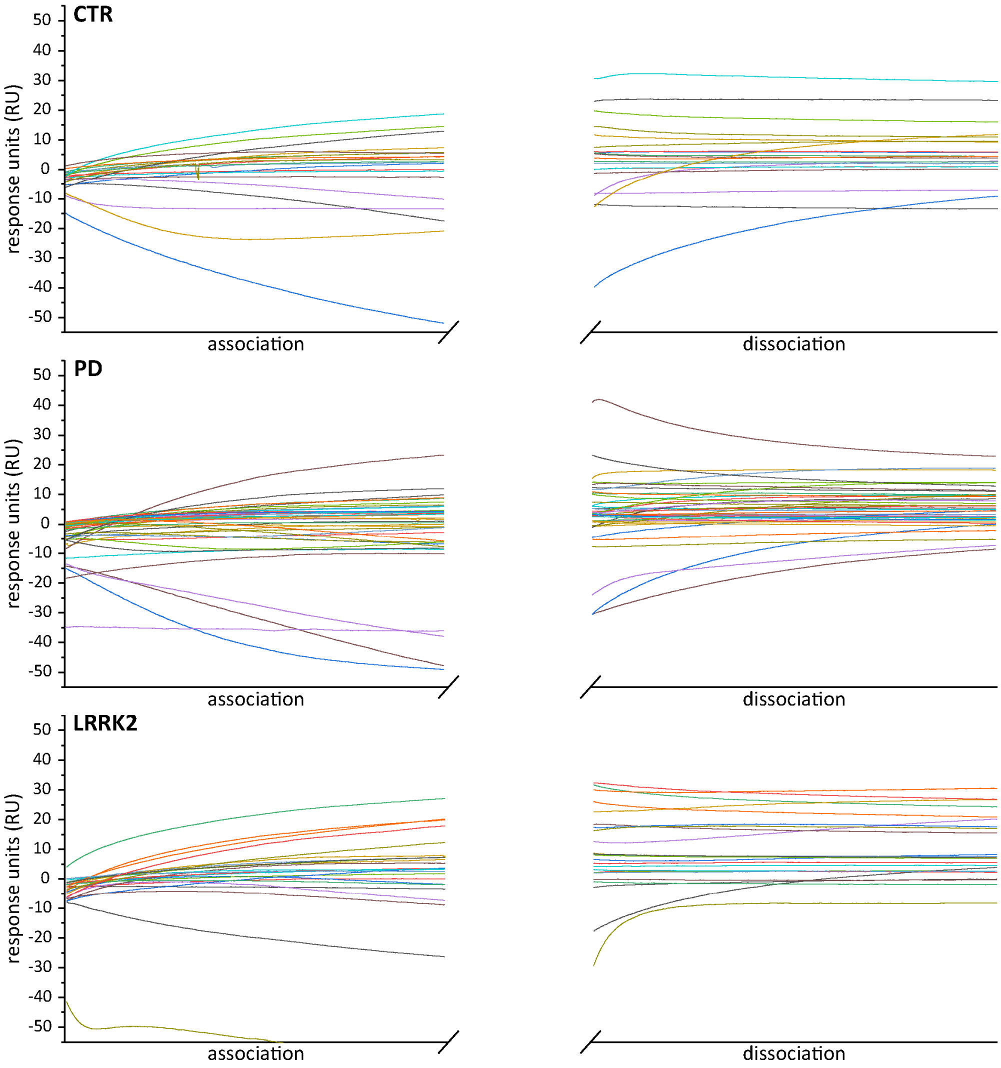

Figure S1. nAbs-α-Syn binding characteristics. Interaction curves of all measured samples are separated for each patient group and for the association and dissociation. Negative trending interactions were rejected for determining the KD.
